# Does Cytomegalovirus Develop Resistance following Antiviral Prophylaxis and Treatment in Renal Transplant Patients in Kuwait?

**DOI:** 10.1155/2011/260561

**Published:** 2011-03-24

**Authors:** Nada Madi, Widad Al-Nakib, Alexander Pacsa

**Affiliations:** WHO Collaborative Centre for AIDS and Sexually Transmitted Disease for EMR, Virology Unit, Department of Microbiology, Faculty of Medicine, Kuwait University, P.O. Box 24923, Safat 13110, Kuwait

## Abstract

The resistance of cytomegalovirus (CMV) to ganciclovir or valganciclovir is a factor in therapeutic failure and disease progression. CMV strains resistant to ganciclovir or valganciclovir have been associated with specific mutations in the UL97 and UL54 genes. Sequencing of both CMV UL97 and UL54 genes was performed to detect the presence of CMV antiviral resistance in six patients who received ganciclovir (and/or valganciclovir) and had prolonged detectable CMV DNA in their blood during antiviral treatment. Sequencing results showed no specific mutations in either UL97 or UL54 gene of CMV and therefore the CMV strains in kidney transplant patients who received ganciclovir either prophylactically or therapeutically were from the wild type. Our results suggest that CMV management and immunosuppression protocols for kidney transplant patients followed in the Organ Transplant Centre, Kuwait, is very effective in reducing the opportunity of developing CMV antiviral resistance.

## 1. Introduction

At present, ganciclovir (GCV) and valganciclovir (VGCV) are the treatment of choice of cytomegalovirus (CMV) in case of infection in solid organ transplant (SOT) recipients [1, 2], and their use has led to a decline in the CMV disease and associated morbidity in SOT recipients [3]. However, with the advent of widespread and prolonged use of antiviral drugs for CMV prophylaxis and the use of immunosuppressive drugs, antiviral-resistant CMV mutants are emerging and are associated with disease progression [4]. Pre-emptive antiviral therapy in transplant patients, on the other hand, is thought to be less likely to lead to the emergence of antiviral drug resistance than is prolonged prophylaxis. Short course of intravenous GCV prophylaxis or treatment have not been associated with GCV resistance in SOT recipient. However, the advent of widespread use of oral GCV for CMV prophylaxis in SOT recipients has led to the selection of GCV-resistant CMV mutants [5].

 Two CMV gene products are implicated in conferring resistance to anti-CMV drugs. One of them is the product of CMV UL97 gene, which is responsible for the first phosphorylation step of GCV, a step necessary for its activity, and the other is UL54 (*pol*) gene product, the primary target of all available antivirals [6]. In this study, our aim was to investigate the presence of drug resistant mutants among CMV-infected kidney transplant patients given GCV (and/or VGCV) whether prophylactically, or therapeutically.

## 2. Materials and Methods

### 2.1. Patients

A total of 54 CMV PCR positive and D+/R+ kidney transplant patients who received kidney transplants between the year 2000 and 2005 at the Organ Transplant Center, Ministry of Health, were involved. Among these patients, 22 were males and 32 were females with a median age of 43 years. These patients received antiviral drugs during the study period. Blood samples were collected every 2–4 weeks unless patients presented with any signs of illness. Drug resistance was suspected when CMV load in the blood failed to decline to undetectable levels despite 2 or more weeks of antiviral treatment. All patients received immunosuppressive treatment depending on their condition to reduce the risk of kidney rejection. The therapeutic protocol was as follows: (1) induction therapy with IL-2 receptor blocker, Simulect (Basiliximab), Zenapax (Daclizumab), and antithymocyte globulin (ATG) for high risk patients only. (2) Maintenance therapy with Prednisolone, Neoral (Cyclosporine), CellCept (Mycophenolate Mofetil), and Prograft (Tacrotimus).

### 2.2. CMV Treatment

The patients received antiviral drugs according to the antiviral chemotherapy protocol which is followed in the Organ Transplant Center; (1) prophylaxis therapy with GCV i.v. (5 mg/kg/b.i.d) or oral VGC (900 mg/day) for 2 weeks. (2) Pre-emptive therapy with GCV i.v. (5 mg/kg/b.i.d) or VGCV (900 mg/day) for 2 weeks. (3) Direct therapy with GCV i.v. (5 mg/kg/twice/day) or VGCV (900 mg/twice/day) for 3 weeks followed by either oral GCV (1g/3 × day) or VGCV (900 mg/day) until clinical resolution.

### 2.3. CMV Monitoring

CMV DNA load in EDTA plasma had been determined prospectively by quantitative real-time polymerase chain reaction (QPCR) using primers and probes that targeted the CMV immediate early gene [7].

### 2.4. Antiviral Resistance Analysis

A 789 bp of a high GC content fragment of UL97 gene was amplified using the following primer pair [8], CPT1088: (Forward) 5^'^-ACG GTG CTC ACG GTC TGG AT-3^'^, CPT1878: (Reverse) 5^'^-GCC ATG CTC GCC CAG GAG ACA GG-3^'^. The amplified region contained the entire known GCV/VGCV-resistance mutations sites (1260–1994 bp), which covers codons 420 to 665. The entire UL54 gene coding for the CMV DNA polymerase, which contains all possible known mutations, was amplified using a set of three primer pairs that were designed by Primer 3 software for primers design (http://frodo.wi.mit.edu/primer3/). The primer sequences are listed in Table 1. After cycle sequencing reactions, sequence analysis of UL97 and UL54 genes of CMV was performed using the automated DNA sequencer CEQ 8000 Genetic Analysis System DNA sequencer (Beckman Coulter, USA) as described by the manufacturer's manual. Blast analysis program (http://www.ncbi.nlm.nih.gov/BLAST) from the National Center for Biotechnology Information (NCBI) was used for the analysis and alignment of the sequences obtained from the automated sequencer with the sequences of wild type CMV strains in the Gene bank (GenBank: FJ616285.1) to identify any of the known mutations in UL-97 or UL54 genes.  Table 2 shows a list of known UL54 mutations conferring GCV resistance in clinical isolates for comparison [9].

## 3. Results

### 3.1. Response of Patients to Antiviral Treatment

The 54 CMV PCR positive kidney transplant patients were treated with either GCV or VGCV alone or a combination of the two drugs for a period ranging between 2-3 weeks and 3 months. During the treatment period, the patients were monitored regularly by measuring viral load in their plasma using the QPCR. Samples from each patient were investigated at the beginning and after the treatment. Forty-eight patients responded well to therapy as indicated by the complete clearance of the virus from the bloodstream at the end of the therapy. However, 6 patients failed to respond well to therapy and were further analysed.  Table 3 shows in detail the effect of the antiviral treatment on the viral load in the plasma samples of these 6 patients. Among the 6 patients, 2 patients were treated according to prophylaxis protocol and 4 were treated according to therapeutic protocol. Patients 1 and 2 had severe CMV disease including pneumonia and graft rejection. In these patients, the viral load at the start of treatment was 4.1 to 4.2 log_10_ copies/mL, and after treatment, the viral load was reduced by 2-folds only (2.1–2.3 log_10_ copies/mL). Patients 3 and 4 had mild symptoms but failed to respond well to therapy. In these patients, the viral load ranged 4.8–4.9 log_10_ copies/mL at the beginning of treatment and showed only slight reduction (3.5–3.9 log_10_ copies/mL) after treatment. Patients 5 and 6, however, had severe form of CMV disease such as diarrhoea, bleeding, and renal failure and failed to respond well to therapy as there were no significant reductions in the viral load after treatment. The viral load ranged 2.8-2.9 log_10_ copies/mL at the beginning of treatment and showed a slight reduction (1.7 log_10_ copies/mL) after treatment.

### 3.2. CMV Antiviral Resistance in Patients Treated with GCV and/or VGCV

#### 3.2.1. Clinical Presentation

**Figure 1:**
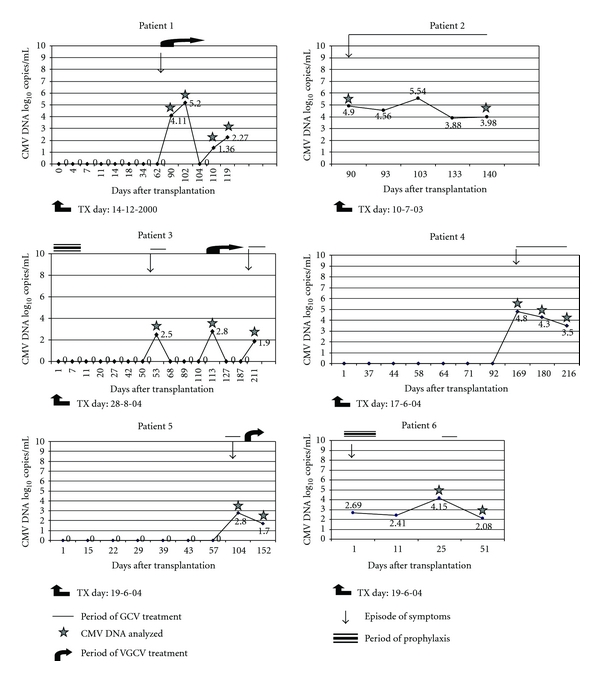
Monitoring of the viral load in kidney transplant patients.

The above 6 patients had CMV-related symptoms and were CMV DNA positive by PCR for more than one-month long period and they did not show significant decreases in viral load despite antiviral treatment. CMV DNA was isolated from samples of these patients and were processed for sequence analysis to determine whether GCV or VGCV treatment had resulted in mutations in the CMV UL97 or UL54 genes. In patient No. 1 (Figure 1), the first positive CMV DNA was obtained 90 days after transplantation. At this time, the patient had fever, leukopenia, and pneumonia. Oral VGCV (900 mg/twice/day) was given for 3 weeks. Although the patient showed considerable reduction in viral load, he failed to clear the virus totally and continued to show CMV DNA in the blood. Viral load was 2.2 log_10_ DNA copies/mL at the end of the treatment. Patient No. 2 (Figure 1) suffered from fever, leukopenia, high creatinine levels, and signs of graft rejection after 90 days posttransplantation. At this time the viral load was 4.9 log_10_ DNA copies/mL. Treatment was started with GCV given as 5 mg/kg/twice/day intravenously for two weeks and was continued with a maintenance dose of 1 g/3 × day orally for three months. During the treatment, the viral load raised slightly (5.5 log_10_ DNA copies/mL) on day 103 posttransplantation. However, after that the viral load dropped but the patient failed to clear the virus and remained CMV DNA positive by day 140 post-transplantation (viral load of 3.9 log_10_ DNA copies/mL). Patient 3 (Figure 1) received an induction ATG immunosuppressive therapy because he showed HLA mismatch and had cadaver kidney, and therefore, he was given VGCV prophylaxis (900 mg/day) for two weeks. Despite this, the patient became CMV positive on day 53 post-transplantation with a viral load of 2.5 log_10_ DNA copies/mL. The patient suffered from thrombocytopenia, fever, and high ALT, and hence he was given 5 mg/kg/twice/day GCV intravenously for two weeks. After the treatment period, the symptoms of the patient were resolved and he became CMV DNA negative in the blood. The patient had a rebound and showed positive CMV DNA again at day 113 post transplantation with CMV DNA load of 2.8 log_10_ DNA copies/mL and was on oral VGCV (900 mg/twice/day) for 3 months. Despite the long treatment period, the patient relapsed at day 211 post-transplantation and had severe systemic infection with leukopenia, renal failure, and bleeding. At this time, CMV DNA could be detected again at relatively lower viral load (1.9 log_10_ DNA copies/mL). In patient 4 (Figure 1), the first CMV DNA positive sample was detected 169 days after transplantation and presented with fever and leukopenia. Treatment was started immediately with GCV (5 mg/kg/twice/day) intravenously for two weeks. However, after treatment, the patient failed to clear the virus and remained positive by day 216 post-transplantation and therefore, was positive for CMV DNA for a total of 124 days. The CMV viral load at the beginning of treatment was 4.8 log_10_ DNA copies/mL, which was reduced to 3.5 log_10_ DNA copies/mL by the end of the treatment. In patient 5 (Figure 1) CMV DNA was detected at day 104 with a viral load of 2.8 log10 DNA copies/mL. At this time the patient presented with fever, leukopenia and diarrhoea. The patient was treated with GCV (5 mg/kg/twice/day) intravenously for 2 weeks and then with oral VGCV (900 mg/twice/day) for one week. Following treatment, the patient remained CMV positive for 95 days with a slight reduction in the viral load by day 152 (1.7 log_10_ DNA copies/mL). Finally, patient No. 6 (Figure 1) received an induction ATG immunosuppressive therapy because he showed HLA mismatch, and therefore, he was given GCV prophylaxis for two weeks. The patient had symptoms of fever, thrombocytopenia, leukopenia, and pneumonia on the day of transplantation. CMV DNA was detected at the day of the transplantation with a viral load of 2.6 log_10_ DNA copies/mL, and after 11 days post transplantation with a viral load of 2.4 log_10_ DNA copies/mL. The patient, however, did not receive treatment immediately, but was treated on day 25 post-transplantation with GCV 5 mg/kg/twice/day intravenously for two weeks. Treatment with GCV did not clear the virus from the blood and the viral DNA was still detectable (2.1 log_10_ DNA copies/mL) by day 52 post-transplantation. This patient with severe disease was not given any other therapeutic such as foscarnet, cidofovir or leflunmide since there is no defined protocol for these drugs in OTC and because of the side effects of some of these drugs. The patient expired before the completion of the follow-up period.

### 3.3. DNA Alterations in UL97 and UL54 Genes

In order to investigate the possible presence of CMV drug resistant virus, viral DNA from a total of 16 CMV strains from the six patients described previously were investigated for mutation by sequencing the UL97 and UL54 genes. These CMV strains were analysed at the beginning, during, and after the treatment. Sequencing of CMV UL97 gene segment of DNA from patient No. 1 did not show any mutations in the nucleotides as compared with the reference strain. However, sequencing UL54 gene segments of CMV strain from the same patients that covered codons 393–576, 593–765, and 820–991 showed five silent mutations that did not result in changes in the amino acid sequence. In patient No. 2, four silent mutations were detected in the gene segment covered codons 585–775 and 820–990 of UL54 gene that did not result in changes in the amino acid sequence. However, no mutations or silent mutation were detected in the UL97 gene. In the case of patient No. 3, no mutations or silent mutation were detected in the UL97 gene. However, five silent mutations were detected in the UL54 gene segments but these again did not result in changes in amino acid sequence. It is worth mentioning that sequence analysis of CMV strain which appeared and collected after five months of treatment also did not show any mutations in both UL97 and UL54 that would result in drug resistance. No mutations or silent mutations were detected in UL97 gene segment from patient No. 4. However, two silent mutations were detected in UL54 gene segments which covered codons 593–765. Moreover, results of UL97 sequence analysis of CMV DNA, isolated from patient No. 5, did not show any alterations in the nucleotides. No nucleotide alterations were detected in UL54 gene segment that covered codons 380–568, while only a single silent mutation detected in gene segment at codon 721. Finally, in CMV DNA isolated from the blood of patient No. 6, no mutations were detected in UL97 gene segments. However, three silent mutations were detected in UL54 gene segment that covered codons 383–572, 593–768, and 815–995.

## 4. Discussion

The goal of this study was to investigate the possibility of the presence of CMV drug resistant mutants after treatment in kidney transplant. Resistance to GCV is associated with mutations in either or both of the UL54 and UL97 genes of CMV. In this study, we have investigated six patients who received GCV and/or VGCV treatment and who did not respond well to antiviral therapy and in whom the viral load did not decrease significantly. Sequencing analysis of DNA of CMV strains collected from these patients at the beginning and after treatment did not reveal any valid mutations in the UL97 and UL54 genes that may confer drug resistance. Among the investigated patients, two were given GCV prophylaxis because they showed HLA-mismatch and had cadaver kidney. We hypothesized that drug resistance may have developed in CMV strains collected from these patients since the possibility of developing drug resistance after prophylaxis therapy is higher than pre-emptive therapy or treatment. Nevertheless, we were unable to detect any mutations in either the UL97 or the UL54 genes of CMV. In none of the samples taken from the 6 patients who showed little or no decline in viral load after treatment were we able to find any mutations in either the UL97 or the UL54 gene. Only silent mutations in UL54 gene were detected. Silent mutations are alterations in the nucleotide sequence that do not generally result in alteration in amino acid sequence of a protein. Boivin et al., have also detected multiple UL54 mutations of unknown significance in clinical strains isolated from SOT recipients [10]. One possible explanation of the absence of drug resistance mutants in our patients is that generally, the incidence of CMV drug resistance in kidney transplant recipients is known to be rather low when compared with the incidence of drug resistance in other solid organ transplant (SOT) and AIDS patients [11]. In a study by Lurain et al., on CMV isolates from SOT patients, it is likely that the incidence of resistance in lung-transplant recipient is higher than other recipient and the incidence of resistance in kidney transplant is rather low [12, 13]. Hantz et al. have also showed that the proportion of SOT patients in their cohort study who suffered treatment failure in the absence of antiviral resistance is high [14]. Furthermore, another study showed that treatment with oral VGCV or intravenous GCV results in similar and low rates of resistance mutations in SOT recipients [10]. So, the possibility of detecting drug resistance in the low number of kidney transplant recipients studied in this project is very low. Usually, drug resistant mutants emerge because of the increased selection of these strains over the wild type as a result of high viral load associated with exposure to potentially inadequate levels of the drug for long periods of time. CMV management and immunosuppression protocols for kidney transplant patients followed in the Organ Transplant Centre, Kuwait, are indeed very effective in reducing the opportunity of developing CMV antiviral resistance. These protocols are based on short-term prophylaxis with GCV or VGCV for only high risk group of patients, and controlled dosage of antiviral drugs for pre-emptive therapy and treatment of CMV infection/reactivation and disease in parallel with adjustable dosage of immunosuppressive therapy. Another factor that may contribute to the emergence of drug resistance is the degree of host immune competence; good evidence showed that rates of CMV-resistant genotypes are higher in more highly immunosuppressed patients such as bone marrow recipients [15, 16]. The question is, what are the factors that may affect the rate of response to antiviral therapy even in the absence of CMV drug resistance? It was observed in our study that CMV DNA in patients with high viral loads tended to persist in the blood for a long period of time (range 70–124 days). However, CMV DNA in patients with relatively lower viral loads persisted for a shorter period (range 46–60 days). Nevertheless, neither group of patients developed resistance based on gene sequencing analysis that we have conducted. Our results are in accordance with other findings, which showed that patients with a higher CMV DNA load more often had a slower treatment response because the time needed to clear DNAemia is related to the amount of virus [17]. Our results are in agreement with those of Humar et al., who demonstrated that delays or failure in clearing the virus are not necessarily related to CMV drug resistance [18]. Van Der Beek et al., have also showed that antiviral resistance was observed infrequently in renal transplant patients who received VGCV and apparently played a minor role in treatment failure [17]. Finally, one of the most important features about the epidemiology of resistance among organ transplant recipients has been the finding that CMV drug resistance is seen almost exclusively in CMV-seronegative recipients of transplants from seropositive donors (D^+^R^−^) rather than in seropositive patients, at least among nonlung transplant recipients [19]. In summary, after the start of therapy, different rates of clearance of CMV are likely to occur due to a combination of different factors including host and virus factors and may not be related only to the presence of drug resistance mutants. Host factors, however, may include the presence of pre-existing immunity to CMV and the degree of exogenous immune suppression.

## Figures and Tables

**Table 1:** Primer sequences for the amplification of UL54 gene.

Primers	Region (bp)	GC content (%)	Size (bp)	Codon
NA-1 (Forward) NA-2 (Reverse)	1794–2394	56%	600	379–579
NA-3 (Forward) NA-4 (Reverse)	2403–3007	62%	604	582–783
NA-5 (Forward) NA-6 (Reverse)	3074–3674	59%	600	806–1006

**Table 2:** UL54 mutations in clinical CMV isolates resistant to GCV [9].

Region	UL54 mutation	Amino acid change
IV	N408D F412C D413E	Asparagine to aspartic acid Phenylalanine to cycteine Aspartic acid to glutamic acid

*δ*-region C	L501F	Leucine to phenylalanine
	L501I	Leucine to isoleucine
	T503I	Threonine to isoleucine
	K503R	Lysine to arginine
	K513E	Lysine to glutamic acid
	P522A	Proline to alanine
	T700A	Threonine to alanine

II	T700A	Threonine to alanine
	M715V	Valine to methionine
	I722V	Isoleucine to valine

VI	V781I	Valine to isoleucine

III	L802M	Leucine to methionine
	A809V	Alanine to valine
	G841A	Glycine to alanine

Other	S676G	Serine to glycine
	G678S	Glycine to serine
	Y751H	Tyrosine to histidine

**Table 3:** Characteristics and outcomes of six patients with clinically suspected drug-resistant CMV.

Patient no.	Age/sex	Clinical symptoms	Drug/regimen	Duration of drug exposure	CMV DNA copy no. (log_10_/mL)	
					At the beginning of therapy	After therapy
1	43/F	Fever, leukopenia, thrombocytopenia, pneumonia	VGCV/treatment	3 weeks	4.2	2.1

2	47/F	Fever, leukopenia, graft rejection	GCV/treatment and maintenance	2 weeks treatment and 3 months maintenance	4.1	2.3

3	60/M	Leukopenia	VGCV/prophylaxis, GCV & VGCV/treatment	2 weeks prophylaxis, 2 weeks + 3 months treatment	4.9	3.9

4	54/F	Fever, leukopenia	GCV/treatment	2 weeks	4.8	3.5

5	55/F	Fever, leukopenia, high creatinene, diarrhoea	GCV & VGCV/treatment	3 weeks	2.8	1.7

6	34/F	Fever, leukopenia, renal failure, bleeding	GCV/prophylaxis, GCV & VGCV/treatment	2 week prophylaxis, 3 months treatment	2.9	1.7
